# Choosing Unwisely: Dissemination Needs of Primary Care Providers of Patients with Alzheimer’s Disease

**DOI:** 10.1093/geroni/igab046.2440

**Published:** 2021-12-17

**Authors:** Lee Lindquist, Aylin Madore, Stephanie Miller, Theresa Rowe, Sara Bradley

**Affiliations:** 1 Northwestern University Feinberg School of Medicine, Chicago, Illinois, United States; 2 DBC Pri-Med, LLC, Boston, Massachusetts, United States

## Abstract

Choosing Wisely is a well-known campaign to disseminate evidence-based clinical practices to providers and patients to drive care decisions, with geriatrics recommendations released in 2013. In December 2019, we aimed to determine what the dissemination needs of primary care providers were towards these recommendations. We developed common clinical scenarios with follow-up survey questions, relative to the care of people with Alzheimer’s disease (AD) and utilizing Choosing Wisely geriatrics recommendations. The survey was distributed online to a national cohort of providers. Providers were also asked to rate their confidence level and rationale for clinical choices. Results were analyzed used mixed methodology, with constant comparative analysis utilized for qualitative responses. Nationally from 41/50 states, 211 providers responded, 72% female, with occupations of physician (36%, 77), advanced practice nurse (50%, 106) and physician assistant (13%, 28), with family practice (63%, 142) and internal medicine (20%, 43) the most prominent fields. Results revealed erroneous geriatric practices, including 1.)checking urinalysis for mental-status changes (55%, 116), 2.)treating asymptomatic bacteria with unnecessary antibiotics (59%, 124), 3.)placement of gastric tubes in end-stage dementia (11%, 23). Qualitative analysis of rationale for incorrect responses revealed knowledge misconceptions (e.g.feeding tube would help avoid aspiration). Confidence levels were high among providers as 75.9% rated themselves as above average, yet did not correlate to clinical errors. Choosing Wisely geriatrics recommendations are not being followed by some providers. Highly confident providers made errors similar to lower confident providers. New ways to disseminate geriatric recommendations are needed to improve the care of patients with AD.

